# Mindfulness: Implementation and evaluation of an intervention program for people with alcohol dependence

**DOI:** 10.1192/j.eurpsy.2023.1197

**Published:** 2023-07-19

**Authors:** R. Costa, N. Rosa, R. Lopes

**Affiliations:** 1 Centro Hospitalar Universitário de Coimbra; 2 Unidade de Alcoologia de Coimbra; 3 UCP Enfermagem de Saúde Mental e Psiquiátrica, Escola Superior de Enfermagem de Coimbra, Coimbra, Portugal

## Abstract

**Introduction:**

The treatment of the person with alcohol dependence allows the possibility of a self-determined alcoholic abstinence and reducing the consequences associated with alcohol-related problems at a personal, family, work and social level.

It is important to develop therapeutic strategies that complement the different approaches in the treatment of people with alcohol dependence, enabling them to use effective coping strategies that facilitate the maintenance of their self-determined alcohol abstinence.

In recent years, scientific evidence has emerged that justifies the adoption of mindfulness-based protocols as a complement to various treatments, both for the prevention of relapses and as a treatment enhancer.

**Objectives:**

To train people with alcohol dependence to use Mindfulness;

To promote psychological well-being and positive emotions;

To reduce anxiety;

To evaluate the effectiveness of a Mindfulness-based intervention program.

**Methods:**

The Mindfulness-based intervention program was developed with 2 groups of people hospitalized for the treatment of alcohol dependence (the institution’s treatment protocol is comprehensive and based on self-determined alcohol abstinence). The 1st G (pilot) - 6 people; 2nd G - 5 people), total of 11 participants; 4 sessions (each group), duration 45-60 minutes.

The selection criteria of the participants were evaluated in an interview and defined as follows: being in the first week of the treatment program; self and allo psychic orientation; reduced to moderate anxiety (Zung Self-Assessment Anxiety Scale - EAAZ); existence of motivation for change.

Participants gave informed consent.

In the global assessment used instruments: Psychological General Well-being Scale for the Portuguese population (BEP); Short Version of the Portuguese Scale of Positive and Negative Affect (PANAS-VRP) in the first session (before intervention). In the last session (after the intervention), in addition to the BEP and the PANAS-VRP, the EAAZ was also used.

At the end of each session, an evaluation was carried out using a grid built for this purpose.

**Results:**

As for the general psychological well-being, the BEP, only one participant (pilot group) had a final score lower than the initial one.

With regard to PANAS-VRP, in both groups, there was an increase in positive affection and a reduction in negative affection at the end of program implementation.

With regard to the EAAZ, 7 participants showed a decrease in anxiety after the intervention.

The evaluation grid of each session revealed good participation, good adhesion and positive evaluation.

**Conclusions:**

It is concluded that after the implementation of the Mindfulness-based intervention program there was: an increase in well-being (the higher the score, the greater the state of well-being); increase in positive affection (which remained or increased); decrease in negative affection and decrease in the level of anxiety.

**Disclosure of Interest:**

None Declared

